# ADHD symptomatology in eating disorders: a secondary psychopathological measure of severity?

**DOI:** 10.1186/1471-244X-13-166

**Published:** 2013-06-11

**Authors:** Fernando Fernández-Aranda, Zaida Agüera, Rita Castro, Susana Jiménez-Murcia, Jose Antoni Ramos-Quiroga, Rosa Bosch, Ana Beatriz Fagundo, Roser Granero, Eva Penelo, Laurence Claes, Isabel Sánchez, Nadine Riesco, Miquel Casas, Jose Manuel Menchon

**Affiliations:** 1 Department of Psychiatry, University Hospital of Bellvitge-IDIBELL, Barcelona, Spain; 2 CIBER Fisiología de la Obesidad y Nutrición (CIBERobn), Instituto Salud Carlos III, Barcelona, Spain; 3 Clinical Sciences Department, School of Medicine, University of Barcelona, Barcelona, Spain; 4 Department of Psychiatry, University Hospital of Vall d´Hebron, Barcelona, Spain; 5 CIBER Salud Mental (CIBERSAM), Instituto Salud Carlos III, Barcelona, Spain; 6 Department of Psychiatry and Legal Medicine, Universitat Autonoma de Barcelona, Barcelona, Spain; 7 Departament de Psicobiologia i Metodologia, Universitat Autònoma de Barcelona, Barcelona, Spain; 8 Department of Psychology, University of Leuven, Leuven, Belgium

**Keywords:** Eating disorders, ADHD, Psychopathology, Personality profiles

## Abstract

**Background:**

Attention-deficit/hyperactivity disorder (ADHD) has commonly been described in psychiatric disorders. Although several studies have found positive associations between abnormal eating patterns during childhood and ADHD, there is a lack of studies on ADHD and Eating Disorders (ED). The aims of this exploratory study were 1) to assess the ADHD symptoms level in ED and to ascertain whether there are differences among ED subtypes; 2) to analyze whether the presence of ADHD symptoms is associated with more severe eating disorder symptoms and greater general psychopathology; and 3) to assess whether the ADHD symptoms level is associated with specific temperament and character traits.

**Methods:**

191 female ED patients were included. Assessment was carried out with the EDI-2, ASRS-v1.1, the SCL-90-R and the TCI-R.

**Results:**

The ADHD symptoms level was similar in bulimia, eating disorder not otherwise specified and binge eating subtypes, and lower in anorexic patients. Obsessiveness and Hostility were significantly positively associated with ADHD symptoms. A path model showed that ADHD was associated with high Novelty Seeking and low Self-Directedness, whereas ED severity was influenced by ADHD severity and low Self-Directedness.

**Conclusions:**

Bingeing/purging ED subtypes have a high ADHD symptoms level, also related with more severe eating, general and personality psychopathology.

## Background

Attention-deficit/hyperactivity disorder (ADHD) has its onset during childhood and is characterized by symptoms of impulsivity, hyperactivity and inattention [1]. Despite being known as a child’s disorder, the symptomatology may persist in adulthood up to 50% of cases [2], with prevalence rates ranging from 1.2% to 7.3% [3].

ADHD has commonly been described in several psychiatric disorders, including affective disorders, substance abuse and impulse control disorders [4], as well as a current comorbid condition [5-7]. Although several studies have found positive associations between abnormal eating patterns during childhood and ADHD [4,8,9], even when compared with controls [10-13], there is a lack of studies on ADHD and Eating Disorders (ED).

Few studies have analyzed the relationship between ED and ADHD and found that the main symptoms of ADHD (namely inattention, hyperactivity and impulsivity) are often present in ED patients [14,15]. The results suggested that ED with ADHD exhibited higher binge-purging features and characteristics, such as low self-esteem, impulsive traits [4,8,16] and neurobiological dysfunctions [17]. However, such studies are restricted to small samples or binge-purging patients and obesity groups, highlighting the need for future studies examining the relationship between ED and ADHD.

In view of this criticism, the present study had the following objectives: 1) to assess the current prevalence of ADHD symptoms in ED and to ascertain whether there are differences among ED subtypes; 2) to analyze whether the prevalence of ADHD symptoms is associated with more severe eating disorder symptoms and greater general psychopathology; and 3) to assess whether the prevalence of more ADHD symptoms is associated with specific temperament and character personality traits.

## Methods

### Participants

One hundred and ninety one female ED patients [43 Anorexia Nervosa (AN), 95 Bulimia Nervosa (BN), 29 Eating Disorders Not Otherwise Specified bulimic subtype (EDNOS-BN) and 24 Binge Eating Disorders (BED)], with a mean age of 28.3 years (*SD* = 9.5), were included. The AN group consisted of 21 AN restrictive subtype (AN-R), 10 AN binge/purging subtype (AN-BP) and 12 subthreshold AN (s-AN, all DSM-IV-TR criteria for AN present except amenorrhea or severe underweight). Patients were consecutively admitted to an outpatient treatment in our Institution and diagnosed according to DSM-IV-TR criteria by means of SCID-I [18]. The study was approved by the Ethics Committee of our hospital and written informed consent was given by all participants before testing.

For the present analysis, from an initial sample of 235 ED patients, the following cases were excluded: a) males (*n* = 14), as the number of males with these diagnoses was too small for meaningful comparison and b) participants with incomplete data in any questionnaire (*n* = 30), [in the Eating Disorders Inventory-2 (N = 5), in the Symptom Checklist-90-Revised (SCL-90-R) (n = 8) and/or in the Adult ADHD Self-Report Scale (n = 17)]. Since the Adult ADHD Self-Report Scale only comprises 6 items, the missing of one item in this questionnaire was considered criterion of exclusion.

### Assessment

#### Eating Disorders Inventory-2 (EDI-2) [19]

This is a reliable and valid 91-item multidimensional self-report questionnaire that assesses different cognitive and behavioral characteristics, which are typical for eating disorders. The EDI-2 retains the 64 items (grouped into eight scales: Drive for Thinness, Bulimia, Body Dissatisfaction, Ineffectiveness, Perfectionism, Interpersonal Distrust, Interoceptive Awareness, Maturity Fears) of the EDI and adds 27 new items into three provisional scales: Asceticism, Impulse Regulation, and Social Insecurity. All of these scales are answered on a 6-point Likert scale, and provide standardized subscale scores. This instrument was validated in a Spanish population [20] with a moderate mean internal consistency of α = 0.63.

#### Adult ADHD Self-Report Scale (ASRS-v1.1) [21]

ASRS-v1.1 was used as a severity indicator of self-reported (current) ADHD symptoms in adulthood. ASRS-v1.1. comprises the 6 out of 18 most predictive items of the Adult ADHD Self-Report Scale (ASRS) [22]. ASRS is a self-administered scale with appropriate psychometric properties [23] based on the DSM-IV criteria and adjusted to reflect ADHD symptoms as seen in adults [23]. The Spanish adaptation of the ASRS was used for rating symptom frequencies on a 5-point Likert scale (0–4) [24]. The total score represents the sum of all responses so that a higher score indicates more symptoms of ADHD.

#### Symptom Checklist-90-Revised (SCL-90-R) [25]

This test contains 90 items and helps measure 9 primary symptom dimensions: Somatization, Obsession-Compulsion, Interpersonal Sensitivity, Depression, Anxiety, Hostility, Phobic Anxiety, Paranoid Ideation, and Psychoticism. In addition, it includes three global indices, which are a global severity index (GSI), designed to measure overall psychological distress; a positive symptom distress index (PSDI), designed to measure the intensity of symptoms as well as a positive symptom total (PST), which are reports of self-reported symptoms. The Global Severity Index can be used as a summary of the test. This scale has been validated in a Spanish population [26], obtaining a good mean internal consistency of α = 0.75.

#### Temperament and Character Inventory-Revised (TCI-R) [27]

The TCI-R [27] is a 240-item, 5-point Likert-scale, reliable, and valid questionnaire that measures, as in the original TCI version [28], four temperament (Harm Avoidance, Novelty Seeking, Reward Dependence, and Persistence) and three character dimensions (Self-Directedness, Cooperativeness, and Self-Transcendence). The performance of the Spanish version of the original questionnaire [29] and the revised version have been documented. The scales in the latter showed an excellent internal consistency of α = 0.87.

### Procedures

All patients were evaluated and diagnosed at the ED Unit of the Bellvitge Hospital. Two semi-structured face-to-face interviews were conducted by psychologists and psychiatrists of the Unit. The first one provided information about current eating disorder, antecedents and other data of interest about the patient. The second interview, with the SCID I [18], covered eating disorder, lifetime prevalence of impulsive behaviours and additional information on family history of eating disorders [30]. The questionnaires mentioned above were administered during the second session when anthropometric measurements were also taken.

### Statistical analysis

Data was analyzed with SPSS19 and STATA12 for Windows. First, the comparison of the total ADHD score between ED diagnosis subtypes was carried out with Analysis of Variance including age and BMI as covariates (ANCOVA).

Next, multiple linear regressions also adjusted by age and BMI explored the associations between the total ADHD score (criterion) and six different set of predictors: eating disorders measures (frequency of bingeing, vomiting, laxatives), EDI-2 scale scores, EDI-2 total score, general psychopathology by means of SCL-90-R scale scores and SCL-90-R global indices, and TCI-R personality scale scores. These analyses were carried out for the total sample as well as for the AN and [BN + EDNOS + BED] groups separately. Moreover, the measure for ADHD has been defined as the dependent variable and as incomes/predictors a large set of independent variables (the ENTER procedure was selected to include all the scales of the same questionnaire in the same model) has been included. This procedure allows the obtaining of the specific contribution of each scale adjusted to the presence of the others. But the adjustment of the multiple regressions was not possible considering the EDNOS and BED groups separately due the low sample size. AN subtype samples were also too small for meaningful comparisons. Anyway, the most prevalent subtype in the AN sample was the restrictive one (21 AN-R, 10 AN-P and 12 subthreshold AN).

Finally, based on the theoretical Cloninger Biosocial model of personality [28], that postulated that Novelty Seeking and impulsive related psychiatric disorder are linked to dopaminergic activity and in recent literature neurobehavioural studies of impulsivity (17), Structural Equation Modeling (SEM) was conducted for testing the path between personality traits (TCI-R), ADHD total score and the intensity of ED (EDI-2 total scale), and global psychopathology (SCL-90-R scores). Robust method of estimation was used and adequate model fit to the data was considered for non-significant results in chi-square test (*p* > .05). Comparative Fit Index (CFI) was higher than 0.90, and Root Mean Square Error Approximation (RMSEA) was lower than 0.06 [31].

## Results

### Clinical and socio-demographic characteristics

Most of the patients were single (68.9%) and employed (73.3%), 21.5% reported primary studies, 47.5% secondary education, and 31% university studies. Table 1 includes the descriptive characteristics of the total sample as well of the ED diagnosis subtypes. With regard to socio-demographic data, significant differences were found in age. Differences also emerged for the variables duration of the disorder, number of previous treatments, and as expected, current BMI.

**Table 1:** Descriptive characteristics of the sample

	**TOTAL ( *N * = 191)**		**AN ( *n * = 43)**		**BN ( *n * = 95)**		**EDNOS ( *n * = 29)**		**BED ( *n * = 24)**		***p***
Age (yrs.)	28.3	(9.51)	25.7	(9.8)	28.6	(8.4)	26.4	(10.3)	34.1	(9.9)	.003
Onset (yrs.)	20.2	(7.75)	18.6	(6.9)	20.1	(7.3)	20.0	(6.8)	23.9	(11.3)	.136
Duration (yrs.)	7.5	(6.55)	5.5	(5.6)	8.4	(6.5)	5.5	(5.8)	8.9	(8.1)	.048
Num. previous treatments	0.40	(0.81)	0.31	(0.6)	0.38	(0.85)	0.77	(1.1)	0.14	(0.35)	.043
Menstruation (yrs.)	12.3	(1.79)	12.1	(1.7)	12.4	(1.9)	12.5	(1.6)	12.3	(1.9)	.747
Body mass index	23.6	(7.39)	17.2	(1.4)	24.3	(6.6)	21.5	(2.1)	35.8	(5.0)	.000

*Mean* (*standard deviation*). *ANanorexia*, *BNbulimia*, *EDNOSeating disorder not otherwise specified*, *BEDbinge eating disorder*.

### ADHD symptoms level

The ADHD questionnaire scores were statistically different between ED subtypes, with AN patients showing significantly lower ADHD scores (M = 9.30). Equal means were found for bulimia, EDNOS, and binge eating (adjusted M = 11.95, M = 11.94 and M = 11.27).

### Association between ADHD, ED and General Psychopathology

Table 2 presents the results of the five multiple regression models valuing the associations between ADHD symptoms, ED symptomatology, general psychopathology, and ED psychopathology, after adjusting by age, in the total and different ED groups. Our results showed a significantly positive association between frequency of binge eating episodes and ADHD symptoms in the total sample, as well as in the BN + BED + EDNOS group.

**Table 2:** **Multiple regression models of different sets of predictors on ADHD scores for the overall sample** (**left**) **and for ED subtype groups** (**centre and right**)

	**Total ( *N * = 191)**				**AN ( *n * = 43)**				**BN + EDNOS + BD ( *n * = 148)**			
**Compensatory behaviours**	***B***	***p***	**CI 95%**		***B***	***p***	**CI 95%**		***B***	***p***	**CI 95%**	
Frequency of binge eating	0.27	.**002**	0.10	0.43	−0.74	.201	−1.90	0.42	0.23	.**010**	0.06	0.41
Frequency of vomits	−0.01	.863	−0.11	0.09	0.24	.057	−0.01	0.50	−0.05	.353	−0.16	0.06
Frequency of laxatives	0.03	.432	−0.05	0.12	0.03	.755	−0.18	0.25	0.03	.567	−0.06	0.11
SCL-90-R subscale scores												
Somatization	0.24	.625	−0.72	1.20	1.74	.164	−0.75	4.22	−0.17	.757	−10.26	0.92
Obsessive - compulsive	2.07	<.**001**	1.04	3.10	0.56	.620	−1.72	2.84	2.15	<.**001**	0.98	3.33
Interpersonal sensitivity	0.49	.394	−0.64	1.62	1.55	.228	−1.02	4.11	0.00	.997	−1.31	1.30
Depressive	0.29	.639	−0.92	1.50	0.13	.938	−3.15	3.40	0.34	.605	−0.97	1.66
Anxiety	−0.52	.392	−1.70	0.67	−0.01	.997	−3.69	3.68	−0.12	.857	−1.41	1.17
Hostility	0.75	.**040**	0.03	1.46	−0.19	.823	−1.89	1.51	0.87	.**039**	0.04	1.70
Phobic anxiety	0.23	.597	−0.63	1.10	−1.67	.113	−3.76	0.42	0.45	.354	−0.50	1.39
Paranoid Ideation	0.04	.942	−0.98	1.05	−0.80	.434	−2.85	1.26	0.44	.475	−0.78	1.67
Psychotic	−0.69	.271	−1.91	0.54	1.00	.382	−1.30	3.31	−1.26	.082	−2.69	0.16
SCL-90-R total scores												
PST score	0.06	.203	−0.04	0.18	0.11	.202	−0.06	0.27	−0.04	.609	−0.21	0.12
GSI score	−0.31	.888	−4.7	4.05	−2.17	.586	−10.2	5.85	3.88	.236	−2.56	10.3
PSDI score	2.42	.127	−0.70	5.54	3.25	.268	−2.61	9.11	−0.48	.841	−5.23	4.27
EDI-2 scores												
Drive for thinness	0.04	.476	−0.08	0.17	0.01	.954	−0.24	0.25	0.10	.230	−0.06	0.25
Body dissatisfaction	0.05	.286	−0.04	0.14	0.00	.970	−0.25	0.24	0.03	.533	−0.07	0.14
Interoceptive awareness	0.16	.**023**	0.02	0.29	0.35	.052	0.00	0.71	0.14	.077	−0.02	0.29
Bulimia	0.03	.585	−0.09	0.16	−0.37	.152	−0.90	0.15	0.04	.568	−0.10	0.18
Interpersonal distrust	−0.12	.171	−0.29	0.05	−0.24	.318	−0.71	0.24	−0.08	.395	−0.27	0.11
Ineffectiveness	0.03	.653	−0.11	0.17	0.22	.375	−0.28	0.73	0.00	.984	−0.15	0.15
Maturity fears	0.03	.581	−0.08	0.14	0.06	.689	−0.24	0.35	0.02	.723	−0.10	0.15
Perfectionism	−0.16	.**029**	−0.31	−0.02	−0.16	.393	−0.54	0.22	−0.14	.100	−0.31	0.03
Impulse regulation	0.03	.662	−0.10	0.16	−0.12	.504	−0.47	0.24	0.10	.213	−0.06	0.25
Asceticism	−0.04	.695	−0.25	0.17	−0.03	.938	−0.82	0.76	−0.08	.456	−0.31	0.14
Social insecurity	0.18	.067	−0.01	0.37	0.15	.649	−0.50	0.79	0.14	.188	−0.07	0.35
EDI-2 total	0.04	<.**001**	0.03	0.05	0.04	.**004**	0.01	0.06	0.04	<.**001**	0.03	0.05

In bold, significant coefficients. Results adjusted by covariates age and BMI.

Among general psychopathology (SCL-90-R), only the Obsessive-compulsive and Hostility subscales were significantly and positively associated with the ADHD questionnaire score, both in the total sample as well as in the BN + BED + EDNOS group. In the AN cohort, no significant associations were found between the ADHD total score and the SCL-90-R scores.

Regarding to ED symptomatology (EDI-2), some significant associations with ADHD symptoms emerged for the total sample. First, the higher EDI-2 total score and EDI-2 Interoceptive awareness score, the higher the ADHD symptoms score. A negative association appeared between the EDI-2 Perfectionism score and the ADHD symptoms scale score. Considering the AN sample, a positive association was found between the EDI-2 total score and the ADHD scale score. Finally, for the BN + BED + EDNOS group a significant association appeared between the EDI-2 total score and the ADHD symptom scale score.

### Personality traits and ADHD

Multiple regressions valuing the predictive accuracy of personality traits (TCI-R scores) on ADHD symptoms score (adjusted by age and BMI) for the total sample obtained that higher Novelty Seeking and lower Self-Directedness were related to higher ADHD scores. Lastly, in the AN subsample, Reward Dependence and Self-Directedness were negatively associated with ADHD symptoms, while Cooperativeness was positively associated (See Table 3).

**Table 3:** **Multiple regression model valuing the association between personality and ADHD**, **for the overall sample** (**left**) **and for ED subtypes** (**centre and right**)

	**Total ( *N * = 191)**				**AN ( *n * = 43)**				**BN + EDNOS + BD ( *n * = 148)**			
	***B***	***p***	**CI 95%**		***B***	***p***	**CI 95%**		***B***	***p***	**CI 95%**	
TCI-R scores												
Novelty seeking	**0**.**05**	.**014**	**0**.**01**	**0**.**10**	0.07	.073	−0.01	0.14	0.03	.205	−0.02	0.08
Harm avoidance	0.03	.209	−0.02	0.08	−0.07	.119	−0.17	0.02	0.04	.140	−0.01	0.09
Reward dependence	−0.02	.395	−0.06	0.02	−**0**.**11**	.**004**	−**0**.**18**	−**0**.**04**	0.00	.961	−0.05	0.05
Persistence	−0.02	.169	−0.05	0.01	−0.03	.387	−0.09	0.04	−0.03	.099	−0.06	0.01
Self-directedness	−**0**.**06**	.**003**	−**0**.**10**	−**0**.**02**	−**0**.**17**	<.**001**	−**0**.**25**	−**0**.**08**	−0.03	.192	−0.08	0.02
Cooperativeness	0.02	.276	−0.02	0.07	**0**.**16**	<.**001**	**0**.**08**	**0**.**24**	0.00	.855	−0.05	0.05
Self-trascendence	0.02	.312	−0.02	0.06	−0.01	.764	−0.09	0.06	0.04	.090	−0.01	0.10

In bold, significant coefficients. Results adjusted by covariates age and BMI.

### Path model to explain personality and clinical outcomes

**Figure 1:**
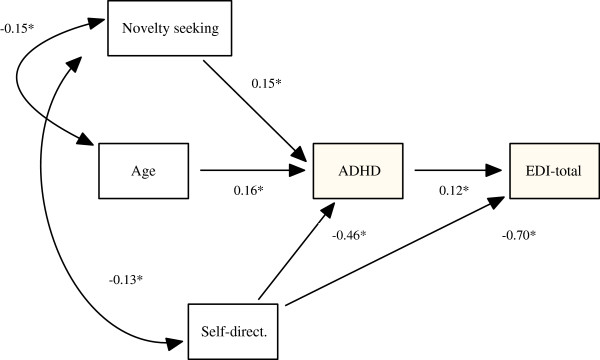
**Structural Equation Model for the path between TCI-R, ADHD and EDI-total scores.**

One structural model was generated to explain the paths between the EDI-2, SCL90-R and TCI-R scales, and ADHD total score jointly. This model included the TCI-R Novelty Seeking and Self-Directedness scales and age as predictors, and the total ADHD score and the EDI-2 total scale as outcomes (Figure 1). Age was included into the model to achieve a better adjustment and due to the strong association observed between this variable and Novelty Seeking and ADHD. This model achieved a good fit (χ^2^ = 4.57, *p* = 0.21, CFI = 0.99, and RMSEA = 0.05) and the total predictive accuracy was very good (R^2^ = 0.585). Results of this model indicated that high scores on Novelty Seeking increase the ADHD symptoms score, while high scores on Self-Directedness decrease ADHD and EDI-2-total scores. High scores on ADHD also are positive associated with the EDI-2-total scale score. A partial mediational path was observed for Self-Directedness, ADHD, and EDI-2-total scores. A complete mediational path emerged between Novelty Seeking, ADHD, and EDI-2-total scores: a high score on Novelty Seeking increases ADHD, and this last score is also positively associated with EDI-2-total.

## Discussion

The current study assessed the ADHD symptoms level in ED patients and ascertained differences among ED subtypes. Moreover, we evaluated the association among the severity of ADHD symptoms and eating disorder symptomatology, general psychopathology and personality.

### ADHD symptoms level in ED subtypes

Our findings support the hypothesis that the ADHD symptoms level in ED patients differs between subtypes. In accordance with previous studies [14,15], our results showed a high frequency of ADHD symptomatology in ED patients. In line with other authors [4,11,32], our findings showed that the more impulsive groups (namely BN, BED and EDNOS subtypes) presented a higher ADHD symptoms level compared to the AN group. The underlying reduced impulse control, a common characteristic of bingeing ED subtypes, might be a shared characteristic with the ADHD population [33]. Accordingly, Pagoto et al. [34] found that poor inhibitory control, associated with deficits in executive functions, may be the main cause of overconsumption, eating without hunger and binge eating patterns. Another possible explanation for this high comorbidity between bulimic disorders (BD) and ADHD is that both disorders share common genetic risk factors. Several polymorphisms of genes and neurotrophic factors have been associated with BN [35] and with ADHD across life span [36]. In the same line, genetic variants of the serotonergic system have been found to be related with BD and ADHD [36-38]. Finally, cognitive features such as rigidity and perfectionism observed in AN patients [8,39] might explain the lower prevalence of ADHD symptoms in this sample. According to this hypothesis clinical reports document perseverative and rigid thinking styles in patients with AN [40,41].

### Association between ADHD, ED and general psychopathology

Our second hypothesis, that the severity of ADHD symptoms is associated with severity in ED symptoms and greater general psychopathology, was supported by our findings. Our results showed a positive association between the frequency of binge eating episodes and ADHD symptoms in the total sample, as well as in the BN, BED and EDNOS group. These findings are in agreement with previous research [11] and suggest that inattention and impulsivity may trigger eating disorders, especially binge eating behaviours. However, these findings are not consistent with a previous study [15] which found that ADHD severity symptoms were not related to the severity of ED symptoms.

Regarding eating symptomatology, our results showed associations between ADHD symptoms and EDI-2 scores. In the total sample, a positive association was found between the EDI-2 total score, the Interoceptive awareness subscale and ADHD symptomatology, which may suggest that an altered Interoceptive awareness combined with the EDI-2 total and with a poorer self directive personality, increases comorbid psychopathology, which is also associated with ADHD symptoms [42].

Another emergent finding was that perfectionism and ADHD symptomatology presented a negative association, i.e. the lower the perfectionism of a subject, the higher the number of ADHD symptoms. This finding goes along with the ED literature, since a perfectionism pattern and a disposition towards over-control are much more related to restrictive ED behaviors such as presented in anorexia, than the binge eating/purging conducts presented in binge eating/purging EDs [43].

Additionally, previous studies in ADHD literature reported that obsessive-compulsive and hostility are frequently found in ADHD patients [2,36]. Correspondingly, both features are also present in patients with eating disorders and are positively associated with the increasing of the severity of the disorder. In our study we found a positive relation between Hostility and obsessive-compulsive patterns and ADHD symptoms, which re-iterates that both disorders have similar comorbid psychopathologies [8].

### Personality traits and ADHD

As we know from research, adult ADHD patients share the same spectrum of personality traits as ED patients. Subjects meeting the ADHD diagnosis showed, in comparison with community subjects, higher scores on Neuroticism-Anxiety, Impulsive-Sensation Seeking and Aggression-Hostility in the Zuckerman Kuhlman Personality Questionnaire (ZKPQ) [44]. Our results showed that ADHD symptomatology was associated with some personality scales, ADHD symptoms showed a positive association with Novelty Seeking and a negative association with Self-Directedness. These results are in line with other studies that have found specific links between Novelty Seeking and impulsivity/hyperactivity on one hand, and low Self-Directedness and inattention on the other hand [45,46]. The negative association between ADHD symptoms and Self-Directedness may represent global lack of personal and social maturity and may reflect deficits in effortful control and executive functioning [45], but also to poorer outcome [45,47].

In the AN group, the ADHD symptomatology was associated with high Cooperativeness and lower Reward Dependence and Self-Directedness. This personality profile has been described in the literature as the "maladaptative" profile and generally presented with the highest values for ED symptomatology and impulsive behaviors [48].

### Limitations

The present study should be evaluated within the context of several limitations. First, only female patients were included, so future studies should also considerer male ED patients. Second, compared to the binge/purge ED group, the AN group was rather small, which could have hidden some interesting findings. Further studies should include more AN patients to compare both the restrictive versus the binge/purging subtypes. Thirdly, this study is limited by the lack of information regarding psychiatric co-morbidity (mainly affective, anxiety and personality disorders). Finally, we assessed the frequency of adult ADHD symptoms by means of a self-rating screening instrument, which might contribute to an overestimation of ADHD symptoms and might explain the elevated prevalence of ADHD symptomatology in the study sample. In the same way, the presence of childhood ADHD was not assessed, which would have been a prerequisite for an ADHD diagnosis in adulthood. Then future studies focusing on the prevalence of ADHD, rather than the frequency of its symptomatology, should be conducted in ED patients. Personality functioning was also based on self-report measures. Thus, future studies might also include performance-based or neuropsychological tasks (i.e. Stroop task or CPT-II test) in order to shed some light on the impulsive behaviour characterizing both binge/purge ED patients and ADHD patients.

## Conclusions

We added to the limited literature on ADHD in the traditional eating disorders and developed a path model to describe the associations between socio-demographic characteristics, personality, ADHD and ED psychopathology. Based on the findings of our path model, we can conclude that both ADHD and ED symptoms (certainly bingeing, purging type) are driven by high levels of novelty seeking and low levels of effortful control. Intervention strategies that focus on the training of effortful control – certainly under rewarding/novel conditions – can be helpful to learn both ED and ADHD patients to increase response inhibition control, decision making abilities and after all to display a higher capacity of planning their behaviour [49,50].

## Abbreviations

ADHD: Attention-deficit/hyperactivity disorder; ED: Eating disorders; BMI: Body mass index; EDI-2: Eating disorders inventory-2; SCL-90-R: Symptom checklist- revised; ASRS-v1.1: Adult ADHD self-report scale; TCI-R: Temperament and character inventory-revised; BED: Binge eating disorder; BN: Bulimia nervosa; AN: Anorexia nervosa; EDNOS: Eating disorders not otherwise specified; DSM-IV: Diagnostic and statistical manual of mental disorders 4th edition

## Competing interests

All authors declare that they have no conflicts of interest.

## Authors’ contributions

FFA, ZA, SJM, JARQ, JMM and MC designed the study. ZA, RB, IS and NR collected the patient data. RG and EP performed the statistical analyses. FFA, ZA, RC, SJM, ABF, JARQ, RB, LC and MC wrote the first draft of the manuscript. All authors commented on and approved the final manuscript.

## Pre-publication history

The pre-publication history for this paper can be accessed here:

http://www.biomedcentral.com/1471-244X/13/166/prepub
